# Acute Metam Sodium Poisoning Caused by Occupational Exposure at a Flower Farm — Uganda, October 2016

**DOI:** 10.15585/mmwr.mm6714a2

**Published:** 2018-04-13

**Authors:** Susan Nakubulwa, Joy Kusiima, Daniel Kadobera, Joan N. Mutyoba, Alex R. Ario, Bao-Ping Zhu

**Affiliations:** ^1^Uganda Public Health Fellowship Program, Kampala, Uganda; ^2^Makerere University School of Public Health, Kampala, Uganda; ^3^Center for Global Health, CDC.

On October 25, 2016, media reports alerted the Uganda Ministry of Health to an outbreak of >80 cases of vomiting, syncope, and acute diarrhea among workers at a flower farm in central Uganda; 27 workers were hospitalized. On November 1, an investigation was undertaken by the Uganda Public Health Fellowship Program.* A case-control study found that working inside greenhouse 7, which had been fumigated with the organosulfur compound metam sodium the night of October 13, was strongly associated with illness. Employees who worked in this greenhouse during October 14–21 reported a strong “suffocating” smell in the greenhouse. Investigation revealed that, in violation of safety protocols, workers did not properly cover the soil after fumigation, allowing vapors to become trapped inside the greenhouse. The farm management, unaware of the lapse, failed to inform workers to avoid the vicinity of the fumigation. Respiratory protective measures were not routinely available for workers, which likely contributed to the severity and extent of the outbreak. Although metam sodium is generally considered to be of low risk when used according to manufacturer’s instructions (*1*), occupational exposure in the absence of recommended safety measures can have serious health consequences. The investigation highlighted the importance of identifying potential occupational hazards to workers, as well as establishing safety protocols in occupational settings, training workers at risk, such as pesticide sprayers and flower pickers,^†^ and ensuring enforcement of safety protocols. After this outbreak, the farm management reviewed, revised, and trained the workers on safety protocols to prevent future outbreaks.

## Epidemiologic Investigation and Findings

A case of greenhouse-associated poisoning was defined as the acute onset of shortness of breath, dizziness, syncope, or vomiting in a farm employee during October 2016. Medical records at the farm’s clinic and nearby hospitals were reviewed. Active case finding was conducted among employees, with the assistance of the farm administrators. Descriptive epidemiologic analyses were performed, which informed hypothesis generation regarding potential exposures. From October 1 to 13, approximately one case of illness had been reported daily; the number of cases increased sharply on October 14, when 17 cases were reported. During the next 16 days, the number of cases declined to an average of two per day (Figure).

**FIGURE:**
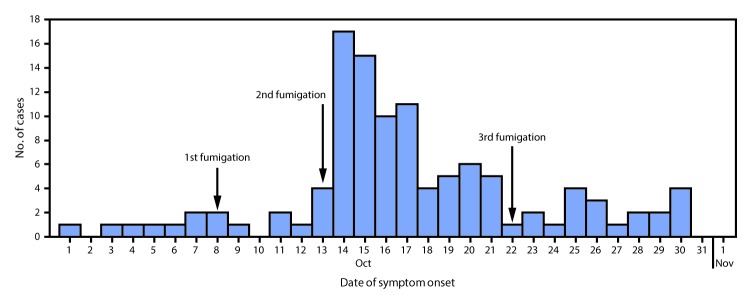
Cases of acute metam sodium poisoning in flower farm employees (N = 110), by date of symptom onset and dates of fumigation of greenhouse 7 — Uganda, October 2016

During the environmental assessment, inspection of the chemical warehouse and review of fumigation protocols and procedures revealed that metam sodium (sodium N-methyldithiocarbamate) had been used to fumigate greenhouse 7 on October 13, the day preceding the sharp increase in the number of reported cases. A Uganda Ministry of Internal Affairs analytical laboratory conducted toxicology testing using triple liquid chromatography mass spectrometry in the recently calibrated triple quadrupole liquid chromatograph mass spectrometer (*2*) for traces of metam sodium or methyl isothiocyanate (a metam sodium degradation product) in the blood of nine symptomatic patients. A case-control study was conducted to compare the exposure histories of 65 case-patients who worked during October 14–21 and 101 controls, selected from asymptomatic employees who had jobs similar to those of case-patients and who worked during the same period. Logistic regression was used to generate odds ratios comparing the odds of exposure to greenhouse 7 for case-patients and controls. The differences in attack rates by sex and occupation were assessed using Pearson’s chi-square test, and statistical significance was defined as p<0.05.

Among the farm’s 562 employees, 110 cases were identified (attack rate = 20%); 104 (95%) cases occurred in women. The mean age of patients was 25 years (range = 17–46 years). The attack rate was higher among women (22%; 104 of 465) than among men (6%; 6 of 97) (chi-square = 13.35; p<0.001), and varied by the nature of work, ranging from 5% among supervisors to 28% among flower pickers, (chi-square = 17.02; p = 0.03) (Table). Overall, 27 (25%) patients were hospitalized and treated with supportive care; no deaths were reported, and all patients fully recovered. The sharp increase in the number of cases on October 14, followed by a gradual decline over the ensuing weeks suggested a continuous common-source toxic exposure. After October 21, incident cases declined to approximately two per day (Figure). The most commonly reported symptoms included dizziness (74%; 81 of 110), shortness of breath (45%; 50 of 110), eye irritation (45%; 50 of 110), and headache (34%; 37 of 110). In the case-control study, 83% (54 of 65) of the case-patients and 34% (34 of 101) of controls reported working in greenhouse 7 during October 14–21 (odds ratio = 9.7; 95% confidence interval = 4.5–21).

**TABLE:** Cases of illness associated with occupational exposure to metam sodium (N = 110) among flower farm workers and attack rates, by sex and job description — Uganda, October, 2016

Characteristic	Total no. of employees	No. of cases (% of all cases)	Attack rate, %
**Sex**			
Male	97	6 (5)	6
Female*	465	104 (95)	22
**Job description**			
Flower picker**^†^**	323	89 (81)	28
Steam boiler attendant	4	1 (1)	25
Scout	10	2 (2)	20
General worker	56	10 (9)	18
Transporter	6	1 (1)	17
Sprayer	15	2 (2)	13
Flower packer	19	2 (2)	11
Quality checker	35	2 (2)	6
Supervisors	20	1 (1)	5
**Total**	**562**	**110 (100)**	**20**

* Chi-square (degrees of freedom = 1) = 13.35, p<0.001.

^†^ Chi-square (degrees of freedom = 8) = 17.02, p = 0.03.

## Environmental and Laboratory Investigations

According to management staff members, the farm’s greenhouses were fumigated with two rounds of metam sodium annually for pest, fungal, and weed control. Each round consisted of fumigation on 3 separate days, approximately 1 week apart. The farm’s safety protocol mandated that the soil be completely covered with plastic sheeting after fumigation, that supervisors double-check to ensure adherence to the protocol, and that the greenhouse be closed for at least 24 hours before anyone could reenter. During the round associated with this outbreak, greenhouse 7 was fumigated on October 8, 13, and 22 (Figure).

Interviews with farm management staff members revealed that after fumigating greenhouse 7 on October 13, workers did not adhere to the safety protocol, poorly covering the fumigated area; this permitted vapors to escape from the soil and become trapped inside the greenhouse. In addition, the mandated postfumigation double-checking by supervisors was not conducted. Also, the requirement for greenhouse closure for at least 24 hours before reentry was not implemented. No environmental testing or air sampling were conducted in greenhouse 7 during the investigation. Interviews of flower pickers revealed that, apart from rubber boots and aprons, no respiratory, ocular or other personal protective equipment was provided by management for use during routine work, although hand-washing facilities were available. Toxicology testing of nine blood samples collected 2–9 days after symptom onset (median = 4 days) did not detect traces of methyl isothiocyanate probably because of chemical degradation over time.

The recommendations to the farm management were to review and revise the safe fumigation protocol, conduct training of workers and supervisors on the protocol, enforce strict adherence to the protocol, and institute mitigation measures should there be an exposure (such as eliminating the hazards promptly and warning workers to stay away from the exposed area). In addition, farm management is currently exploring alternative, less toxic methods for soil treatment, including steaming and biologic methods.

## Discussion

Metam sodium (sodium N-methyldithiocarbamate), a liquid dithiocarbamate, is widely used as a soil fumigant, pesticide, herbicide, and fungicide in agricultural practices (*3*), with relatively low acute toxicity (*4*). However, upon exposure to the environment, the chemical degrades to methyl isothiocyanate, a low melting and powerful lachrymator (*5*). Although toxicology testing of ill patients in this outbreak did not detect traces of methyl isothiocyanate,^§^ the signs and symptoms reported by patients and the sharp increase in the number of cases immediately after fumigation of the greenhouse in violation of the recommended safety protocol suggest that this outbreak was caused by exposure to metam sodium vapors, which escaped from the soil into greenhouse 7.

Respiratory, neurologic, and ocular symptoms have been reported in persons exposed to metam sodium. After 19,000 gallons of metam sodium were spilled into the Sacramento River in northern California in 1991, an outbreak of respiratory and neurologic symptoms was reported in the surrounding community (*6*). In 2002, after a soil-incorporated application of metam sodium, a community outbreak of acute ocular and respiratory illnesses occurred in Arvin, California (*7*). Respiratory effects of metam sodium exposure have been shown to persist for up to 13 months after the initial acute poisoning (*8*). Teratogenicity studies have demonstrated maternal and fetal toxicity in experimental animals such as rats and rabbits after metam sodium exposure (*9*). The U.S. Environmental Protection Agency has identified metam sodium as a B2 (probable human) carcinogen (*9*).

Compared with the outbreak after the Sacramento River spill (*6*,*10*), a higher percentage of patients in this outbreak experienced neurologic symptoms, such as dizziness (74% versus 30%), and syncope (15% versus 0%). This difference might have been because the metam sodium vapors were trapped inside a greenhouse, and the workers, whose typical work shift was 8 hours, were exposed in an enclosed space, whereas after the Sacramento River spill, vapors reached residents in their homes after being dispersed in the wind. After the Sacramento River spill, some persons reported symptoms more than 1 week after the incident (*10*). During the current outbreak, some patients developed symptoms several days after their exposure.

The findings in this report are subject to at least three limitations. First, toxicologic testing did not find evidence of methyl isothiocyanate in the blood samples of the patients tested. Laboratory studies in rats have shown that >85% of orally administered metam sodium was excreted within 24 hours (*9*). However, no published data are available on human metabolism of metam sodium. It is possible that any metam sodium inhaled or absorbed by the patients was fully metabolized by the time the samples were collected. Second, the case definition was broad and included nonspecific symptoms, which might account for the high background rate. On the other hand, because employees were not provided with appropriate personal protective equipment such as masks, the high background rate might represent ongoing chronic exposure to various chemicals, including metam sodium, used in flower farming. Also, the flower farm routinely applied other chemicals for pest control in addition to the seasonal fumigation, including thiovit (micronised sulfur),^¶^ copper oxychloride,** and trigard,^††^ all of which can cause eye, skin, and respiratory tract irritation. These chemicals were not used in greenhouse 7 during the exposure period; however, the effects of these chemicals and their degradants might partially explain the relatively high number of background cases. Finally, air testing was not conducted to assess the level of metam sodium in greenhouse 7 during the outbreak period.

The flower industry is among Uganda’s top income generators. Uganda’s flowers are exported to European Union countries, the United States, and other countries. In 2015, Uganda exported 7,500 tons of flowers, generating $34 million in revenue; a quarter of those flowers were sold to the United States (Uganda Flowers Exporters Association, Performance Statistics, unpublished data, 2015). Ensuring implementation and enforcement of safety protocols for application of fumigants and other pest controls is important in protecting the safety and health of workers in this growing industry.

**Summary**

**What is already known about this topic?**

Metam sodium (sodium N-methyldithiocarbamate) is widely used in agriculture as a soil fumigant, pesticide, herbicide, and fungicide. Acute health effects of metam sodium exposure have been rarely described.

**What is added by this report?**

In October 2016, an outbreak of vomiting, fainting, and diarrhea occurred among employees of a flower farm in central Uganda; 27 employees were hospitalized. Illness was associated with working inside a greenhouse recently fumigated with metam sodium. Safety protocol violations led to this outbreak.

**What are the implications for public health practice?**

This outbreak highlights the importance of establishing, training workers on, and enforcing safety protocols in occupational settings and ensuring that workers are provided with appropriate personal protective equipment.

## Footnotes

* Uganda Public Health Fellowship Program, a collaboration between the Uganda Ministry of Health, Makerere University School of Public Health and CDC is based in the Ministry of Health with the main function of investigating disease outbreaks as part of the National Rapid Response Team.

† https://www.cdc.gov/niosh/topics/hierarchy/.

§ A highly sensitive spectrometer that can detect up to 100 ng/mL was used for toxicology testing.

¶ http://www.herbiguide.com.au/MSDS/MSULP800_53904-0605.pdf.

** http://www.uap.ca/products/documents/2011-CopperSprayPCP19146.pdf.

†† https://www.msdsdigital.com/trigard%C2%AE-msds.
